# Riproximin Exhibits Diversity in Sugar Binding, and Modulates some Metastasis-Related Proteins with Lectin like Properties in Pancreatic Ductal Adenocarcinoma

**DOI:** 10.3389/fphar.2020.549804

**Published:** 2020-11-30

**Authors:** Micah N. Sagini, Karel D. Klika, Andrew Orry, Michael Zepp, Joshua Mutiso, Martin R. Berger

**Affiliations:** ^1^ Toxicology and Chemotherapy Unit, German Cancer Research Center, Heidelberg, Germany; ^2^ Molecular Structure Analysis, German Cancer Research Center, Heidelberg, Germany; ^3^ MolSoft LLC, San Diego, CA, United States; ^4^ Department of Zoological Sciences, Kenyatta University, Nairobi, Kenya

**Keywords:** Pancreatic ductal adenocarcinoma, lactosyl-sepharose binding proteins, cellular lectins, ribosome-inactivating protein, monosaccharides, affinity

## Abstract

Riproximin (Rpx) is a type II ribosome-inactivating protein with specific anti-proliferative activity. It was purified from Ximenia *americana* by affinity chromatography using a resin coupled with lactosyl residues. The same technique facilitated isolation of proteins with lectin-like properties from human Suit2-007 and rat ASML pancreatic cancer cells, which were termed lactosyl-sepharose binding proteins (LSBPs). The role of these proteins in cancer progression was investigated at mRNA level using chip array data of Suit2-007 and ASML cells re-isolated from nude rats. These data compared significant mRNA expression changes when relating primary (pancreas) and metastatic (liver) sites following orthotopic and intraportal implantation of Pancreatic Ductal Adenocarcinoma (PDAC) cells, respectively. The affinity of Rpx to 13 simple sugar structures was modeled by docking experiments, the ranking of which was principally confirmed by NMR-spectroscopy. In addition, Rpx and LSBPs were evaluated for anti-proliferative activity and their cellular uptake was assessed by fluorescence microscopy. From 13 monosaccharides evaluated, open-chain rhamnose, β-d-galactose, and α-l-galactopyranose showed the highest affinities for site 1 of Rpx’s B-chain. NMR evaluation yielded a similar ranking, as galactose was among the best binders. Both, Rpx and LSBPs reduced cell proliferation *in vitro*, but their anti-proliferative effects were decreased by 15–20% in the presence of galactose. The program “Ingenuity Pathway Analysis” identified 2,415 genes showing significantly modulated mRNA expression following exposure of Suit2-007 cells to Rpx *in vitro*. These genes were then matched to those 1,639 genes, which were significantly modulated in the rat model when comparing primary and metastatic growth of Suit2-007 cells. In this overlap analysis, LSBP genes were considered separately. The potential suitability of Rpx for treating metastatic Suit2-007 PDAC cells was reflected by those genes, which were modulated by Rpx in a way opposite to that observed in cancer progression. Remarkably, these were 14% of all genes modulated during cancer progression, but 71% of the respective LSBP gene subgroup. Based on these findings, we predict that Rpx has the potential to treat PDAC metastasis by modulating genes involved in metastatic progression, especially by targeting LSBPs.

## Introduction

Pancreatic ductal adenocarcinoma (PDAC) is a lethal disease with high mortality, which is attributed to its aggressive nature and late diagnosis (Distler et al., 2013). Its incidence is almost equal to fatalities with a 5-year survival rate remaining below 8% over the last decades (Orth et al., 2019). Statistics show that in 2018, there were 458,918 cases of PDAC, of which 432,242 succumbed to the disease (Borazanci et al., 2019). In the United States alone, it was estimated that 56,770 cases would be detected in 2019 and 45,750 would die from the disease (Siegel et al., 2019). At the time of diagnosis, only 15–20% of patients have a chance for long-term survival if they qualify for surgical resection (Hackert, 2018). In addition to surgery, the preferred chemotherapy for PDAC includes modified FOLFIRINOX and gemcitabine and/or nab-paclitaxel regimens (Raufi et al., 2019). To prolong patients' survival, novel drugs are urgently needed for improving the current regimens, which have a limited efficacy.

Rpx is a plant lectin, which showed anti-proliferative activity against a panel of 17 PDAC cell lines (Adwan et al., 2014a). It belongs to the toxic type II ribosome-inactivating proteins, which exhibit affinity for galactose (Voss et al., 2006b). Toxic type II RIPs such as ricin, abrin and viscum lectin I are dimeric proteins with the A-chain linked to the B-chain (lectin-like) by a disulfide bond (Marshall et al., 2011; Schrot et al., 2015; Wang et al., 2016). As found for other type II RIPs, the toxicity of Rpx in tumor cells results from the inhibition of protein synthesis following translational arrest by the A-chain. When Rpx interacts with cancer cells, its B-chain binds to cell surface glycans, thus facilitating entry of the A-chain. In the cell, the A-chain inhibits protein synthesis by depurinating ribosomal RNAs (Bayer et al., 2012a). Besides inhibiting protein synthesis, Rpx was also shown to induce cell death through unfolded protein response (Horrix et al., 2011). Rpx can be purified from the fruits of *Ximenia americana* by affinity chromatography using a resin coupled with lactosyl residues (Bayer et al., 2012b). When evaluated by sequence analysis and molecular modeling, Rpx was found to interact with galactose via its two sub-domains in the B-chain (Voss et al., 2006b). In addition, experiments by carbohydrate microarray demonstrated that Rpx binds to *N-* and *O-* linked glycans, particularly to the bi- and tri-antennary NA2/NA3 and Tn3 structures, which, respectively, bear Galβ1and GlcNAcα moieties (Bayer et al., 2012a). Both, carcinoembryonic antigen (CEA) and mucins are the primary carriers of *N-* (NA2/NA3) and *O-* (Tn antigens) linked glycans (Bergstrom and Xia, 2013; Zhao et al., 2018). Results from our previous studies suggested that carcinoembryonic antigen-related cell adhesion molecule (CEACAM) glycoproteins are targets of Rpx. In line with this observation, Rpx was effective against human (Suit2-007) and rat (ASML) pancreatic cancer cells growing in the liver of nude and immunocompetent rats (Adwan et al., 2014b; Murtaja et al., 2018).

For refining a previously established model based on human Suit2-007 PDAC cells growing in the liver of nude rats (Eyol et al., 2012), the liver as site of secondary growth was compared with that of the primary site. Thus, by comparing the gene expression profiles of Suit2-007 cells growing in the pancreas (primary site) and liver (secondary site) we expected to identify genes that are active in the liver metastatic progression of this cell line. Recently, our group (Sagini et al., 2018) published a description of genes modulated under these conditions. In the present article, we have used these data to further characterize genes, which were modulated by exposure to Rpx.

The isolation of Rpx from *X. americana* was accomplished by affinity chromatography using a lactosyl resin (Bayer et al., 2012b). In another study, this procedure served for isolating proteins from Suit2-007 cell lysates, which were subsequently identified by label-free quantification mass spectrometry (LFQ-MS). The resulting proteins were termed lactosyl-sepharose binding proteins (LSBPs). Characterization by biophysical techniques revealed that they bind simple sugars like galactose (Sagini et al., 2020). This characteristic feature was identical to that of the plant lectin *riproximin* (Rpx), which had been purified by the same method.

In the present study, we aimed at delineating any common features that may exist between Rpx and PDAC derived LSBPs, because they were isolated by a matching method. To this end, we investigated the antiproliferative activity of Rpx and LSBPs against Suit2-007 cells in the absence or presence of galactose, followed by exposure of Suit2-007 cells to Rpx to determine its effect on LSBPs’ gene expression. For achieving the latter objective, we used two approaches to specify those genes, which are supposed to validate the importance of LSBPs in PDAC progression. The first approach was based on using the chip array data of Suit2-007 and ASML PDAC cells growing in nude and BDX rats, respectively, for further specification. The fold change in expression from pancreas to liver served as a measure for the potential involvement of a given gene in cancer progression. The second approach involved using genes from a data set, which had been downloaded from the omnibus public depository (ID: GSE71989). These data contain gene profiles comparing human PDAC and normal pancreatic tissue samples. Again, the fold change in expression between normal pancreatic tissue and PDAC served to identify genes, which potentially contribute to PDAC induction. By these procedures, we wanted to identify LSBPs with importance for PDAC progression.

## Materials and Methods

### Docking Experiments

A homology model of RPX was built using ICM-Pro v3.79 (San Diego, CA, United States) based on the closest homolog template in the Protein Data Bank (PDB 2VLC) (Abagyan et al., 1994; Cardozo et al., 1995; Abagyan et al., 1997). The polypeptide chain was aligned onto the template and then the side-chain torsions were refined using a Biased Probability Monte Carlo (BPMC) optimization method in the Internal Coordinate Mechanics Force Field (ICMFF) (Abagyan and Totrov, 1994; Arnautova et al., 2011). To identify small molecule binding cavities in the refined model of Rpx, we used the ICM PocketFinder method-(Trochon et al., 1996). Five types of docking energy interaction potentials were calculated for each site based on: 1) van der Waals potential for a hydrogen atom probe; 2) van der Waals potential for a heavy-atom probe (generic carbon of 1.7 Å radius; 3) optimized electrostatic terms; 4) hydrophobic terms; and 5) lone-pair-based potential, which reflects directional preferences in hydrogen bonding. Thirteen monosaccharides were docked independently for each site using the ICM-Pro docking method. Each monosaccharide was given a score based on the interaction energy of the pre-calculated docking potentials (Totrov and Abagyan, 1999). During docking the sugar structure is fully flexible and is sampled based on the BPMC method (Abagyan and Totrov, 1994).

### Nuclear Magnetic Resonance Spectroscopy

NMR spectroscopy was done as described before (Sagini et al., 2020). In short, binding was evaluated by line broadening or pertinent chemical shift changes observed in the proton (^1^H) spectrum as well as by the transfer of bulk water magnetization to the ligand, using waterLOGSY pulse sequence. The proton ^1^H NMR spectra were acquired at 298 K with a Bruker Avance II NMR spectrometer. The instrument was equipped with a 5-mm inverse-configuration probe with triple-axis-gradient capability at field strength of 14.1 T operating at 600.1 MH*z* for ^1^H nuclei. The ^1^H NMR spectra were acquired using the 1D NOESY pulse sequence. A pre-saturation of the water signal was applied using standard spectral parameters as for normal ^1^H NMR spectra. WaterLOGSY spectra were also acquired using the pulse sequence of as described by Dalvit (Dalvit, 1996).

### Separation and Identification of Lactosyl-Sepharose Binding Proteins From ASML Cells

ASML and Suit2-007 cells were routinely propagated as described before (Al-Taee et al., 2018; Sagini et al, 2018). For LSBP analysis, cells were lyzed in lysis buffer and isolated by affinity chromatography as described before (Sagini et al., 2020). Then, proteins were analyzed by label free quantification mass spectrometry. Briefly, fractions separated by affinity chromatography were run on a SDS-gel (∼0.5 cm). Protein bands were excised from the Coomassie blue stained gel and digested by trypsin. Sample digests were loaded onto a cartridge trap column packed with Acclaim PepMap300 (Thermo Scientific) and separated by a 120 min gradient (3–40% ACN) on a nanoease MZ Peptide analytical column. Eluted peptides were then analyzed by an online coupled Q-Exactive-HF-X mass spectrometer.

### Cell Proliferation Assay

In this assay, the proliferation of Suit2-007 cells was evaluated in response to Rpx treatment. Suit2-700 cells (4,000 cells/well) were seeded into 6-well plates in complete RPMI1640 medium. The plates were then kept under standard culture conditions. After 24 h, the cells were treated with Rpx (2.5 ng/ml) or LSBPs (500 μg/ml), as well as with lower concentrations, respectively. Following incubation under standard cell culture conditions for 48 h, the anti-proliferative effects were evaluated by adding MTT (3-(4,5-dimethylthiazol-2-yl)-2,5-diphenyltetrazolium bromide) solution (100 μl/well). After another incubation period of 3 h, 100 μl of 2-propanol solution (containing 0.04 N HCL) was added to each well. Then, an Elisa reader measured the absorbance at 540 nm, with 690 nm as the reference wavelength. To evaluate the effect of galactose on Rpx and LSBPs, the same concentrations of Rpx and LSBPs were co-incubated with galactose (500 µM) for 30 min before treating the cells.

### Evaluation of cellular Uptake of Rpx and Lactosyl-Sepharose Binding Proteins by Microscopy and Fluorescence-Activated Cell Sorting Analyses

To evaluate cellular uptake of Rpx and LSBPs, proteins were labeled with the dye Alexa Fluor 647 (Invitrogen, Karlsruhe, Germany) as described previously (Sagini et al., 2020). Briefly, the proteins were separately dissolved (2 mg/ml) in sodium bicarbonate buffer (36 µl of a 0.1 M stock, pH 8.3), and mixed with Alexa Fluor 647 (50 μl). They were incubated at room temperature for 1 h under constant vortexing (350 rpm). Then, the solutions were passed through two separate gel filtration columns (PD10 - Sephadex G-25, GE, Life Sciences, München, Germany) using phosphate buffer (pH 8.0). The colored fractions, which represented labeled Rpx or LSBPs, were collected and concentrated using 10 kDa Amicon filters (Merck KGaA, Darmstadt, Germany). The labeled proteins were sterile filtered and kept at −20°C until further use.

For microscopic evaluation, Suit2-007 (10,000 cells/200 µl/well) were seeded in LabTek glass chambers for microscopy (Thermo Scientific, Germany). After 24 h, the cells were separately treated with Rpx (IC_25_; 0.04 ng/ml) or LSBPs (IC_25_; 3 μg/ml), and incubated under standard cell culture conditions for 1 h. Thereafter, the cells were washed with HBSS and stained with EpCAM (Alexa fluor 488, Thermo Scientific, Germany) or negative control antibodies (5 µl/well). Then, the cells were incubated again for 15 min in the dark. This batch of treated and control cells was used for subsequent analysis by microscopy and FACS. For fluorescence microscope evaluation (Leica systems GmbH, Mannheim), fresh HBSS was added to the cells and images were taken at wavelengths corresponding to 488 and 647 nm, respectively, at 630 fold magnification (oil immersion). Captured images were processed by ImageJ software (NIH-United States).

For FACS analysis, cells were trypsinized and respective aliquots were suspended in FACS buffer. FACS analyses were performed with a FACSCalibur flow cytometer (Becton Dickinson Biosciences, Heidelberg, Germany), with lasers corresponding to green and red colors. Ten thousand events were analyzed, respectively, and processed by Flowing Software.

### Chip Array for Riproximin-Treated Cells

Suit2-007 cells were cultured in complete RPMI media as described previously (Sagini et al., 2018). For Rpx treatment, cells were seeded in 6-well plates (2.5 × 10^5^ cells/well) and kept under standard culture conditions for 24 h. After this period, the plates were treated with 0.312 ng/ml Rpx, corresponding to the IC_50_. The cells were incubated for 48 h under standard culture conditions, after which they were harvested to obtain cell pellets. The isolation of total RNA was performed as detailed in the Fast Gene RNA isolation kit (NIPPON Genetics Co., Ltd.). The concentrations of total RNA were determined by a Nanodrop spectrophotometer, and samples were transferred to the Genomic and Proteomic Core Facility for chip array analysis. For chip array analysis, hybridization of biotin-labeled cRNA samples on Illumina Human Sentrix-12 BeadChip arrays (Illumina, Inc., San Diego, CA, United States) was performed according to the modified Eberwine protocol as detailed elsewhere (Sagini et al., 2018).

### Analysis of Patient Samples Downloaded From Gene Expression Omnibus

GEO2R is a platform freely available for comparing two or more groups of samples in a gene expression omnibus (GEO) series to identify genes that are differentially expressed across experimental conditions. This platform performs comparisons on original submitter-supplied processed data tables using the GEOquery and limma R packages from the Bioconductor project based on the R programming language.

In total, 21 samples representing eight normal pancreatic tissue and 13 pancreatic cancer tissues were downloaded from the gene expression omnibus database (GSE71989). These samples were previously analyzed using the Affymetrix Human Genome U133 Plus 2.0 Array platform and deposited in August 2015 into the GOE database. The samples were analyzed as described in the database. Briefly, the downloaded data was analyzed by the limma R package, which resulted in gene expression profiles with adjusted *p* values (adj.*p*-value i.e., *p* value after adjustment for multiple testing), t-statistics, B-statistics (log-odds that the gene is differentially expressed) and expressed in Log2-fold changes between two experimental conditions). The resulting data was converted to fold-change in expression and uploaded to the IPA (Ingenuity Pathway Analysis) program for comparative analysis with two other data sets (Rpx treated PDAC cells as well as samples from the PDAC liver metastasis rat model).

### Statistical Analysis

Chip array and proteomics data were analyzed as described previously (Sagini et al., 2020). MaxQuant software (version 1.6.0.16) was used for proteomics data. For chip array, the empirical Bayes method and R program were used for data analysis (*p* < 0.05). For PDAC patient samples, adj.P.Val’s were generated for multiple samples (Smyth, 2004; Tyanova et al., 2015). Further analysis of filtered data sets was performed by IPA (core analysis *p* < 0.01). Identification of common and unique genes was performed using Venn diagram maker (online at http://bioinformatics.psb.ugent.be/webtools/Venn/). The data on antiproliferative activity were expressed as mean with standard deviation; they were presented using the program GraphPad Prism six and analyzed by two way ANOVA for significance. *p* values <0.05 were considered significant.

## Results

### Modeling and Docking Experiments With Riproximin and Monosaccharides

**FIGURE 1:**
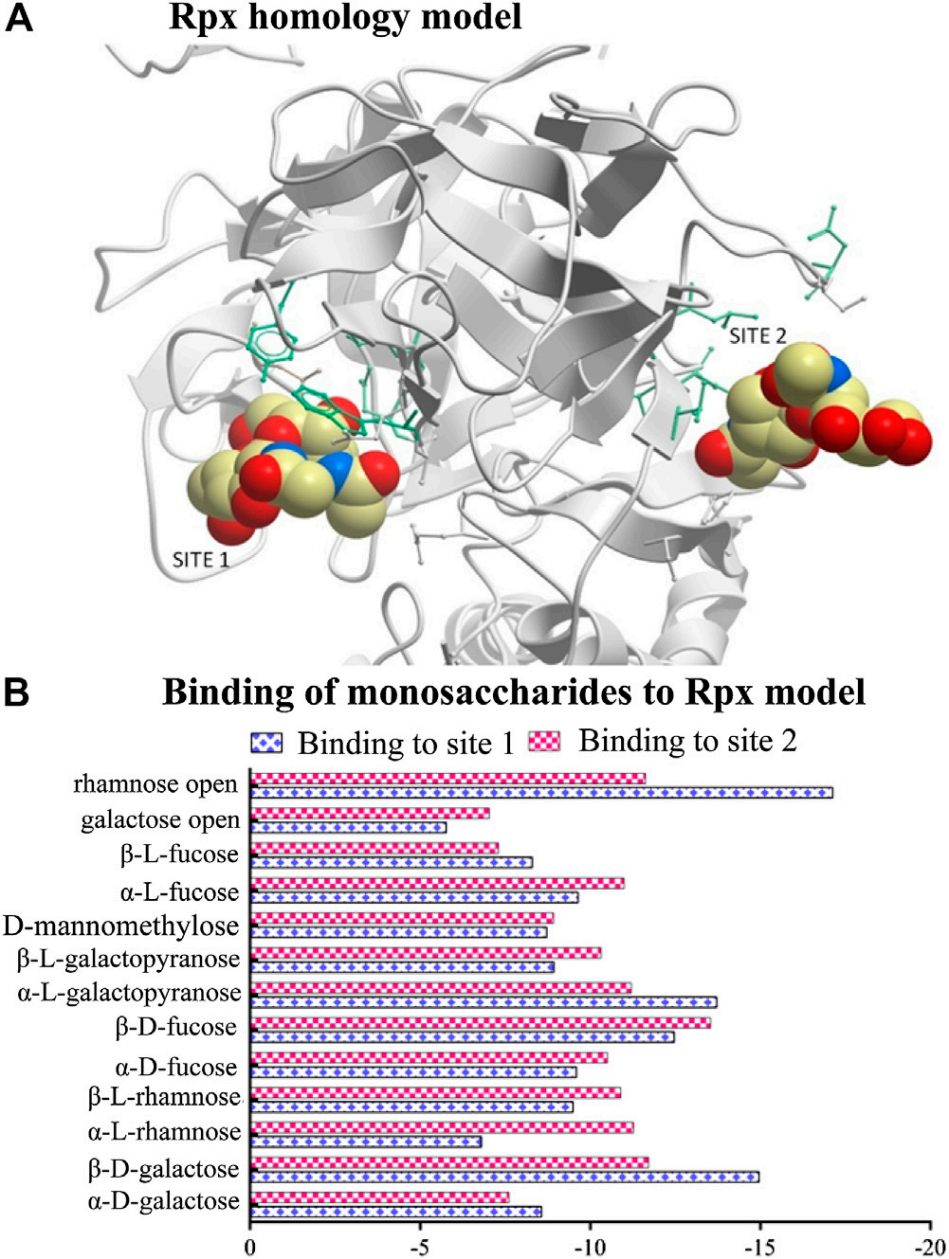
Modeling of riproximin’s structure and its affinity for selected monosaccharides. **(A)** Represents a homology model of the B-chain of Rpx showing two sugar binding sites. The model was built from the closest homology template derived from the Protein Data Bank (PDB 2VLC) using ICM-Pro v3.79 software (Molosoft LLC, San Diego CA). **(B)** Represents a profile of energy scores obtained from docking experiments. In total, 13 monosaccharides were docked to the two binding sites of Rpx. Low energy scores indicate high affinity.

A homology protein model with two binding sites representing the B-chain of Rpx (Figure 1A) was built using the program ICM-Pro v3.79 – (Molosoft - LLC, San Diego, CA, United States) (Abagyan et al., 1994). In total, 13 monosaccharides were selected from the PubChem database and docked against each sugar-binding site to evaluate their energy scores. The resulting energy scores from these experiments represent the binding strength of each monosaccharide, with low values indicating high affinities. The docking experiments were based on 1) van der Waals potential for a hydrogen atom probe; 2) van der Waals potential for a heavy-atom probe (generic carbon of 1.7 Å radius); 3) optimized electrostatic term; 4) hydrophobic terms; and 5) lone-pair-based potential, which reflects directional preferences in hydrogen bonding (see Supplementary Tables S1A,B).

All sugar structures except three (open-chain rhamnose, β-d-galactose, and α-l-galactopyranose) showed comparable energy scores against each binding site (Figure 1B). Interestingly, open-chain rhamnose showed the highest affinity of all tested monosaccharides with a higher affinity for site 1 than for site 2. The next most affine monosaccharides were β-d-galactose, and α-l-galactopyranose, which showed the same preferences for binding sites 1 and 2. The negative energy scores were determined by unique amino acids that characterize each binding site. Site1 was surrounded by nine residues, viz. P275 (proline), Q277 (glutamine), T291 (threonine), P330 (proline), R333 (arginine), N363 (asparagine), T364 (threonine), L369 (leucine), and N457 (asparagine), and site 2 by seven residues, viz. D271 (aspartic acid), K414 (lysine), W416 (tryptophan), T420 (threonine), Y447 (tyrosine), M543 (methionine), and I553 (isoleucine).

### Binding of Monosaccharides to Riproximin by Nuclear Magnetic Resonance Spectroscopy

The binding of monosaccharides to Rpx was evaluated by NMR spectroscopy as described previously (Sagini et al., 2020). The three monosaccharides evaluated for binding to Rpx were d-galactose and the two 6-deoxyhexoses l-rhamnose and l-fucose. These sugars exist predominantly in two pyranose-ring forms, viz. the α- and β-anomers, for which binding was evaluated. The furanose-ring and open-chain forms of each sugar were not analyzed since their content was too low for effective evaluation.

**FIGURE 2:**
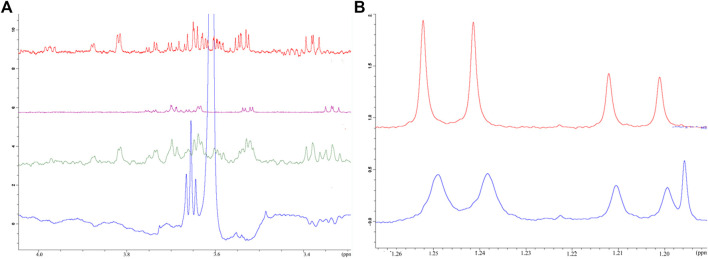
Affinity of selected monosaccharides to riproximin by NMR spectroscopy. **(A)** Reveals the binding of galactose and fucose to Rpx as evaluated by waterLOGSY NMR spectra. Control experiments, i.e. in the absence of Rpx, are represented by galactose (red trace), fucose (pink trace) and both sugars (green trace) together. The waterLOGSY spectrum in blue shows the negative resonances for the ring protons of galactose and fucose in the presence of Rpx, thus indicating the binding of these sugars to Rpx. **(B)** Represents the binding of l-fucose to Rpx as demonstrated by line broadening in the ^1^H NMR spectrum. The methyl doublet signals of l-fucose show clear line broadening due to binding to Rpx (bottom trace in blue). The more intense lines of the doublet at 1.25 ppm of the β-anomer are broadened to a greater extent than the lines of the doublet at 1.21 ppm of the α-anomer, thereby indicating greater affinity of the β-anomer to Rpx. A control spectrum is also presented (top trace in red) (N.b. The additional line in the spectrum with Rpx is from ethanol).

Initial evaluation of the binding of the monosaccharides to Rpx was based on observed line broadening, a technique which is commonly used for determination of protein–ligand interaction. The affinities of both d-galactose and l-fucose for Rpx were moderate as evident by clear broadening of the signals in their 1H NMR spectra. The comparative line broadening of the anomeric H-1 signals in the case of d-galactose and l-fucose, as well as the methyl signals in the case of l-fucose (see Figure 2B) indicated stronger binding for the β-anomer than for the α-anomer, for both d-galactose and l-fucose.

Binding was also confirmed by waterLOGSY spectra (see Figure 2A and Supplementary Tables S1A,B), which also established the order of binding of the anomeric forms of the two sugars. It was revealed that the β-anomer of d-galactose has a higher affinity for Rpx than the β-anomer of l-fucose, and that the α-anomer of d-galactose shows a slightly higher affinity for Rpx than the α-anomer of l-fucose. Though the affinity of l-rhamnose to Rpx was not evident from the ^1^H spectrum, a water-LOGSY spectrum confirmed nevertheless the very weak binding of both, the α- and β-anomers of l-rhamnose to Rpx (data not shown). By waterLOGSY, β-l-rhamnose exhibited a higher affinity for Rpx than α-l-rhamnose (see Supplementary Table S1), though the difference was minimal. Thus, evaluation of these three sugars revealed that the α- and β-anomeric pyranose-ring forms had differential affinities, with the β-anomer always showing higher affinity than the α-anomer. Thus, the NMR evaluation of affinity for the six sugar species to Rpx shows the following ranking: β-d-galactose > β-l-fucose > α-d-galactose > α-l-fucose >> β-l-rhamnose > α-l-rhamnose. Moreover, based on the signal intensity changes in the waterLOGSY spectra, it was evident that the binding epitope encompasses in each case the entire molecule.

### Galactose Reduced the Activity of Riproximin and Lactosyl-Sepharose Binding Proteins *In Vitro*

**FIGURE 3:**
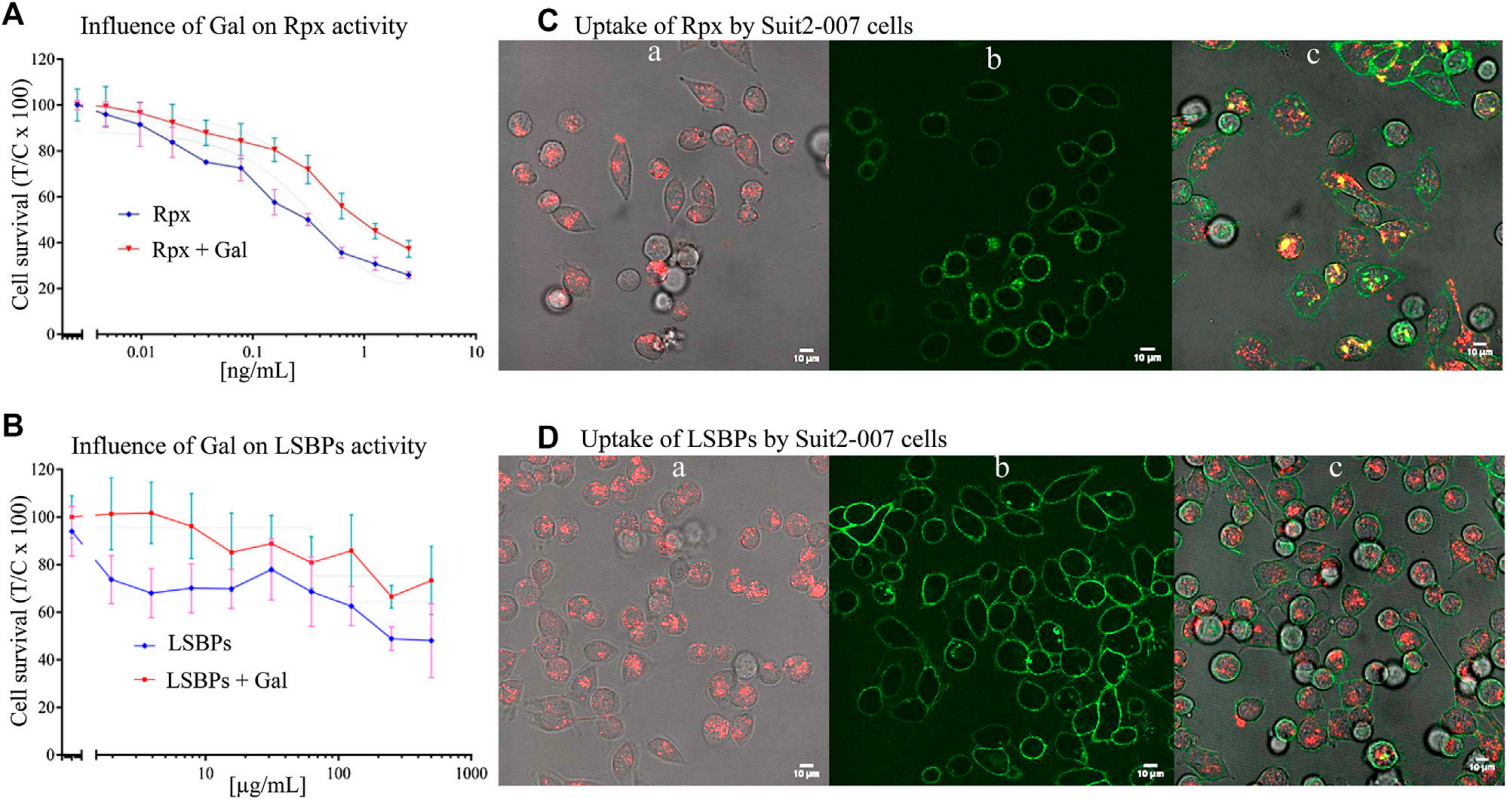
Anti-proliferative effect of riproximin and lactosyl-sepharose binding proteins. **(A,B)** Represent the anti-proliferative effects of Rpx and LSBPs at 48 h, respectively, in Suit2-007 PDAC cells (blue lines). In addition, these effects were antagonized by prior incubation with galactose (red lines). **(C,D)** (a, b, and c) represent microscopic images of Suit2-007 cells, that were exposed to Rpx as well as LSBPs. These proteins had been labeled with the dye Alexa fluor 647 and are indicated by red color. In addition, the Suit2-007 cells were exposed to an antibody against EpCAM, linked to the dye Alexa fluor488, which is visualized by green color. The three corresponding images (a, b, and c) show aspects of the individual colors and their combination, respectively. Images were taken by a fluorescence microscope at 630-fold magnification (oil immersion).

To evaluate the effect of galactose on the activities of Rpx and LSBPs, clean (i.e., galactose free) fractions of these proteins were used to determine their cellular activity. For Rpx, concentrations ≤2.5 ng/ml were sufficient to reduce cell proliferation, which was accompanied by apoptotic cell death (Figure 3A). In the case of LSBPs, concentrations ≤500 μg/ml were required to reduce cell proliferation (Figure 3B). The IC_50s_ for Rpx and LSBPs were attained at 0.312 ng/ml and 375 μg/ml, respectively. A co-exposure of Rpx or LSBPs with galactose (at 500 µM) significantly reduced the activity of both treatments by 15–20% (*p* < 0.001).

### Intracellular Uptake of Riproximin and Lactosyl-Sepharose Binding Proteins by Tumor Cells

For evaluating cellular uptake of Rpx and LSBPs, the proteins were labeled with the fluorescent dye Alexa Fluor 647 (red) and the antibody against EpCAM (coupled to Alexa Fluor 488, green), as described previously. Suit2-007 cells were treated with Rpx (IC25; 0.04 ng/ml) or LSBPs (IC_25_; 3 μg/ml), and incubated for 1 h under standard conditions. After this period, treated cells were evaluated by fluorescence microscopy for intracellular localization of Rpx and LSBPs. Red dots in the cells represent labeled Rpx (Figures 3Ca) or LSBPs (Figures 3Da) following cellular uptake. Figures 3C,D, b represent labeling of the cell surface with the EpCAM antibody. Finally, Figures 3C,D, c show both colors and demonstrate the presence of intact PDAC cells as well as the uptake of labeled proteins into these cells. Cellular uptake of Rpx and LSBPs presumably resulted from the binding of these proteins to cellular glycans before entering the cells. Once taken up, Rpx caused induction of apoptosis, as seen from cells after 24 h of exposure, showing presence of blebbing, chromatin condensation and condensed nuclei, whereas LSBPs did not exert a similarly strong cellular reaction. Thus, prolonged incubation of Suit2-007 with Rpx caused induction of apoptosis (results not shown).

**FIGURE 4:**
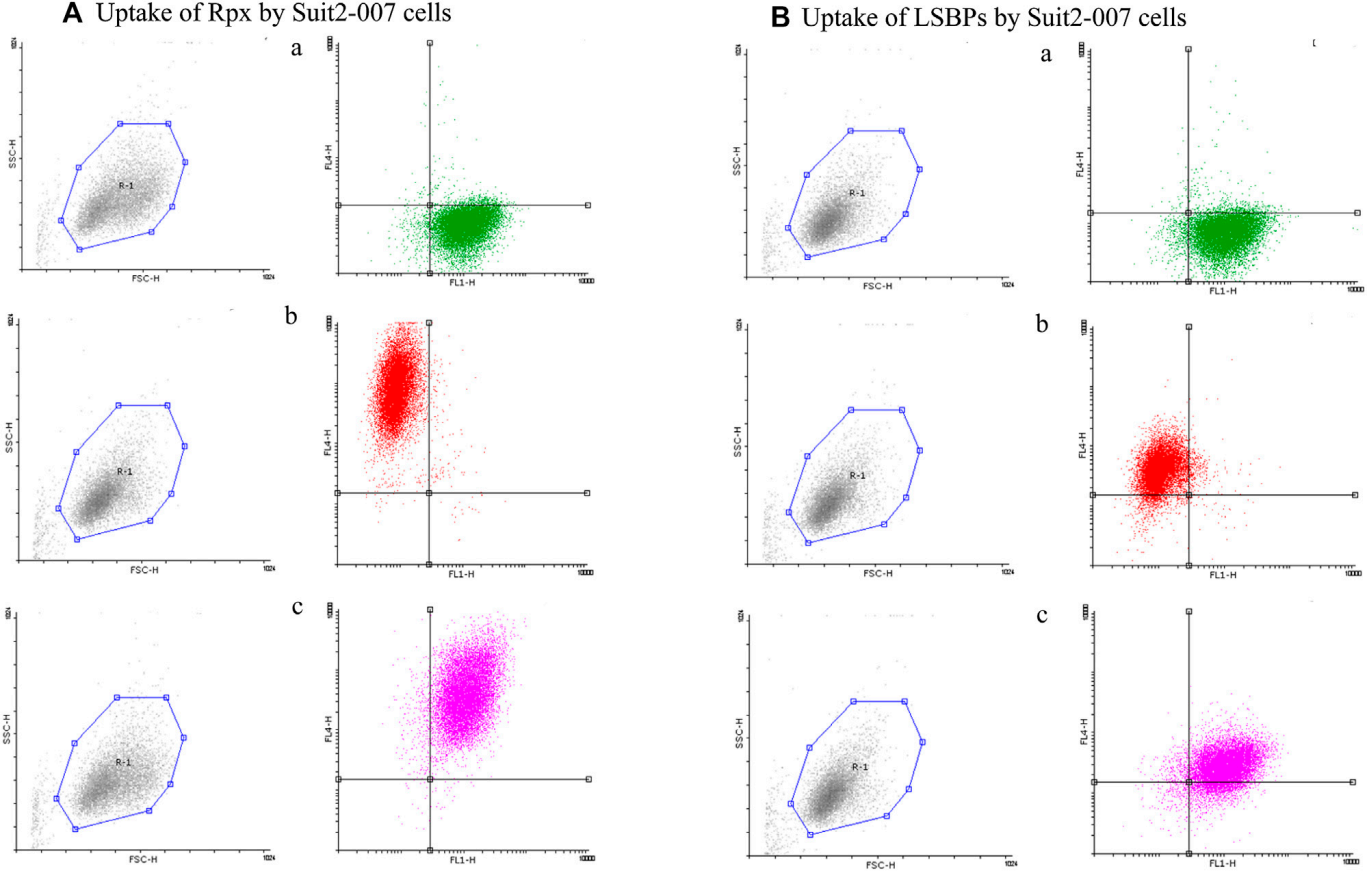
Cellular uptake of riproximin and LSBPs by FACS. **(A,B)** Represents the FACS analysis of an uptake experiment, in which Suit2-007 cells had been exposed to riproximin or LSBPs. Both, riproximin and LSBPs had been labeled with the dye Alexa Fluor 647 and an antibody against EpCAM, labeled by the dye Alexa Fluor 488. **(A)** Shows Suit2-007 cells, which had been exposed to riproximin, and **(B)** those, which had been exposed to LSBPs. The corresponding letters a, b, and c are indicative of the antibody stained cells (green), Alexa Fluor labeled (red) riproximin or LSBPs, and a combination of the two colors, respectively.

In addition, cells of the same batch were analyzed by FACS for quantifying the uptake. As shown in Figures 4A,B, a, Suit2-007 cells were uniformly stained for EpCAM, as represented by the green color. When comparing the uptake of labeled proteins (red color) after 1 h exposure, riproximin was incorporated by one to two orders of magnitude more efficiently (Figures 4Ab) than LSBPs (Figures 4Bb). By using both colors, the uptake was seen in the vast majority of intact cells (Figures 4A,B, c).

### Modulation of Lactosyl-Sepharose Binding Proteins by Riproximin *In Vitro*

**FIGURE 5:**
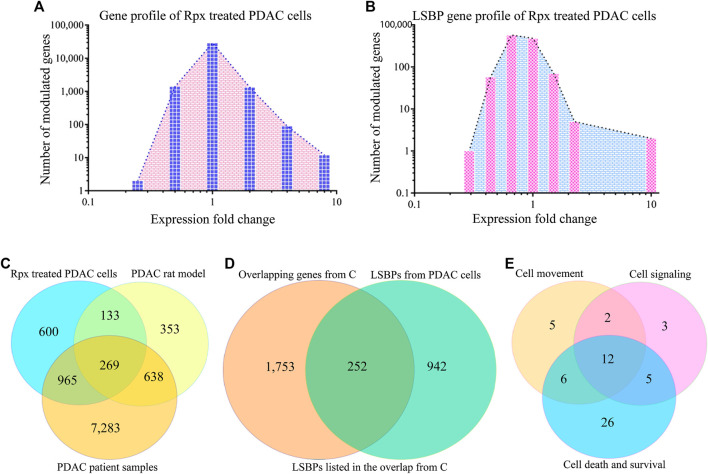
Analysis of riproximin-induced changes in gene expression. **(A)** Represents the mRNA expression of Suit2-007 cells following Rpx treatment at 48 h. **(B)** Shows the mRNA expression for the subgroup of LSBP genes, which were extracted from the data shown in **(A)**. **(C)** Represents a Venn diagram analysis for all modulated genes (*n* = 15,257) from the three matching data sets, i.e., from Rpx-treated Suit2-007 cells, the PDAC rat model and PDAC patient samples. **(D)** Is a Venn diagram analysis for genes listed in at least two or more data sets, as shown in **(C)**: These genes (*n* = 2,481) were related to all identified LSBPs (*n* = 1,194). **(E)** Represents a Venn diagram analysis for 68 of the 269 LSBP genes shown in **(D)**, which were obtained following IPA evaluation. The analysis focused on three functional annotations, viz “cell movement”, “cell signaling” and “cell death and survival”.

After exposing Suit2-007 cells to the IC_50_ of Rpx (0.312 ng/ml), treated and control cells were evaluated for modulation of gene expression by chip array. The relative changes from all cellular genes (23,000) are summarized in Figure 5A. The modulation of the subgroup of LSBP genes (*n* = 1,194) is shown in Figure 5B. For all genes, there was a tendency of more genes being significantly (±1.5 fold) up-regulated (*n* ^up^ = 1,269; 5.5%) than being down-regulated (*n* ^down^ = 1,146; 5.0%). In slight contrast, LSBPs were more often down-regulated (*n* ^down^ = 120; 10.1%) than up-regulated (*n* ^up^ = 94; 7.9%).

When considering all genes (2,415) that were modulated at IC_50_ in response to Rpx exposure, 214 genes (8.9%) corresponded to LSBPs (Supplementary Figure S1A). Next, for establishing the ratio of LSBP genes that are associated with cancer progression in the rat model, the respective chip array data were analyzed. Based on this analysis, we compared the changes in expression of Rpx-treated genes with those obtained from tumor growth in different environments (liver vs. pancreas) (Sagini et al., 2018). From 1,639 genes, which were modulated when comparing expression levels in liver and pancreas, 175 genes (10.7%) corresponded to LSBPs (Supplementary Figure S1B).

Based on the overlaps shown in Supplementary Figures S1A,B and Table S1 gives an overview on LSBP genes. These genes show significant modulation of expression in Suit2-007 cells, in response either to Rpx or because of the differential growth conditions from liver and pancreas organ environments in nude rats. For characterizing their cellular distribution, the (extra-) cellular compartments are given together with the numbers of respective LSBPs. Obviously, the presence of LSBPs is not confined to a single cellular structure, but they are distributed through all compartments, with a relative peak in the cytoplasm.

When identifying common genes between the LSBPs derived from Supplementary Figures S1A,B, which are analyzed in Table 1 (389 genes in total), 49 LSBP genes emerged, corresponding to 12% of genes showing modulation of expression in both, Rpx treated and PDAC rat model derived LSBPs.

**TABLE 1:** Cellular distribution of LSBP genes in Suit2-007 PDAC cells^a^

Cellular location	Rpx treated PDAC cells^b^		PDAC rat model (liver/pancreas)^c^	
	Up-regulated^d^	Down-regulated^d^	Up-regulated^e^	Down-regulated^e^
Extracellular	00	14	14	08
Plasma membrane	14	15	11	12
Cytoplasmic	45	69	48	44
Nucleus	30	17	16	17
Other locations	05	05	01	04
Sum	94	120	90	85

LSBPs, lactosyl-sepharose binding proteins; PDAC, Pancreatic Ductal Adenocarcinoma.

a The distribution of LSBP genes, which are modulated in expression, is shown for Suit2-007 cells exposed to riproximin as well as for cells from the same cell line after re-isolation from liver and pancreatic organ environments.

b Suit2-007 cells were exposed to riproximin at IC 50 concentration for 48 h.

^c^Suit2-007 cells were implanted into RNU rats by orthotopic implantation (pancreas) or via the portal vein (liver). The tumor cells were re-isolated and analyzed by micro-array, as described in (Sagini et al., 2018).

d The gene expression ratio of treated vs. control cells was used for defining upregulation or downregulation (±1.5 fold).

e The gene expression ratio between Suit2-007 cells re-isolated from liver and pancreas was used for defining upregulation or downregulation (±1.5 fold).

In addition, a data set was used, which derived from human PDAC and normal pancreatic tissues. From all genes (*n* = 48,000) detailed, 9,154 genes showed at least a ±1.5 fold modulated expression in PDAC compared to normal pancreatic tissue. When comparing these genes with those from the rat model, 892 genes were significantly modulated in both data sets (Supplementary Figure S1C), corresponding to 54.4% of the rat model (progression) genes and 9.8% of the patient sample genes. Finally, when combining all three data sets with significant modulation (Rpx, rat model and patient samples *n* = 10,241) and searching for the presence of LSBPs, 812 genes corresponding to LSBPs (68%) were found (Supplementary Figure S1D).

A formal analysis of overlapping genes from all three data sets is given in Figure 5C. When concentrating on overlapping genes only (*n* = 2,005) and determining the overlap from the three data sets with LSBPs, the corresponding number of genes was 252, which represents 12.6% of all genes present in at least two data sets (Figure 5D).

To further explore the relevance of the 252 LSBP genes in metastatic progression, we performed IPA analysis. This analysis resulted in gene listings, which were assigned to various functional annotations. From these, we selected genes under the terms “cell movement”, “cell signaling” and “cell death and survival” for further analysis. The selection of these annotations was based on their relevance in metastasis, significant Z-score and corresponding *p*-value. This analysis identified 59 genes (19.4%), which are related to the above mentioned annotations. The resulting Venn diagram shows the distribution of these genes (see Figure 5E and Supplementary Table S2).

To determine whether LSBPs found in human PDAC Suit2-007 cells were also present in cancer cells of rat origin, an additional experiment with a PDAC rat cell line (ASML) was performed. Mass spectrometry analysis of ASML lysates separated by affinity chromatography resulted in 1,949 LSBPs. The gene IDs of these LSBPs were then searched in data of a chip array with ASML cells performed previously (Al-Taee et al., 2018), for relating these proteins with their respective mRNA expression levels. The chip array data had been performed on cells re-isolated from the liver of immunocompetent rats, in which the ASML cells were allowed to grow for different periods (3, 6, 16, and 21 days). The modulation of LSBPs is given in Supplementary Figure S2A. A Venn diagram analysis of 1,194 LSBPs from Suit2-007 and 1,949 LSBPs from ASML cells showed that 741 genes were present in both cell lines (Supplementary Figure S2B). From these genes, we searched those, which are involved in the three different functional annotations (“cell movement”, “cell signaling” and “cell death and survival”), based on the IPA analysis described above. With a Venn diagram analysis, we identified 45 LSBP genes in ASML cells from 67 genes identified in Suit2-007 cells (Supplementary Table S2 and Figure S3C). The 45 overlapping genes in the Supplementary Table S2 are marked with an asterisk.

**FIGURE 6:**
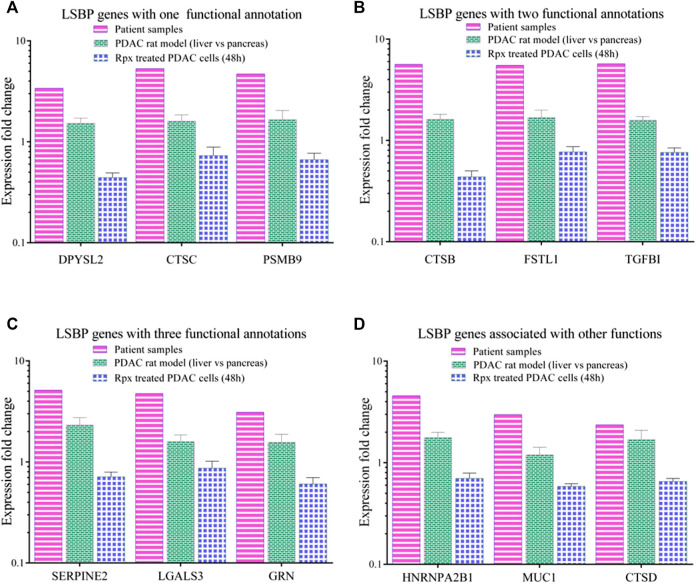
Comparison of modulated LSBP genes selected from three functional annotations. **(A–C)** Represent bar graphs of LSBPs genes selected from **(E)**, which were involved in one or more functional annotations. The colors represent the respective data sets from which they originate, i.e. human samples (pink), rat model (green) and Rpx-treated Suit2-007 cells (blue). The genes selected are related to one (A), two (B) or three (C) functional annotations as detailed in **(D)**. **(D)** Represents genes related to different functions.

From Figure 5E and Supplementary Table S2, three genes from each functional annotation, respectively, were selected for display as shown in Figure 6. These genes were significantly up-regulated (1.5 fold in at least one data set) in patient samples as well as in liver environment of the Suit2-007- rat model. Remarkably, that increase was more distinct in patient-than in rat samples. However, when Suit2-007 cells were exposed to Rpx, these genes showed significant down-regulation (1.5 fold). Genes linked to one, two, or three annotations are shown in Figures 6A–C, respectively. The genes include dihydropyrimidinase like 2 (DPYSL2), cathepsins (CTSB/C/D), proteasome subunit beta 9 (PSMB9), follistatin like 1 (FSTL1), tumor growth factor beta1 (TGFB1), Serpin Family E Member 2 (SERPIN2), galectin 3 (LGALS3) and granulin precursor (GRN). In addition, Figure 6D represents genes, which we considered potentially relevant, although they are associated with other functional annotations. They include heterogeneous nuclear ribonucleoprotein A2/B1 (HNRNPA2B1), mucin 1 (MUC1) and cathepsin D (CTSD). The relevance of these genes was largely confirmed by their presence among the LSBPs of ASML cells.

**FIGURE 7:**
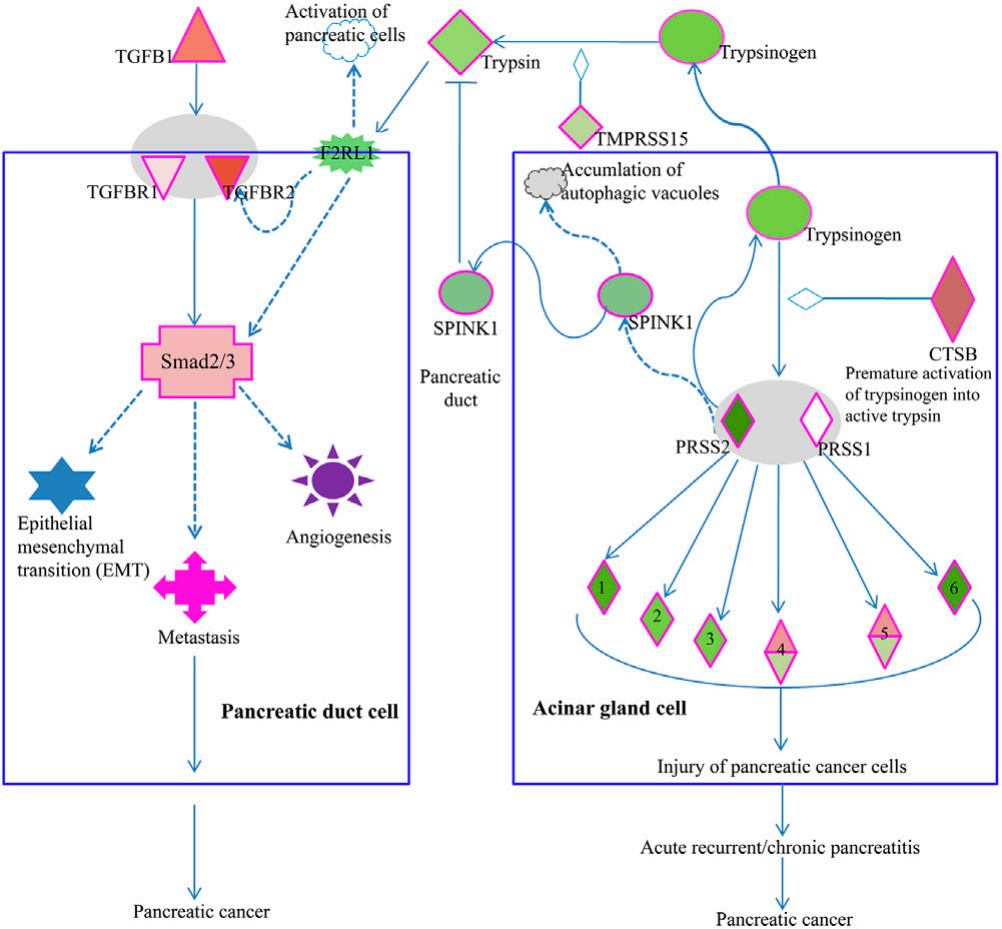
Elements of the SPINK1 pathway are involved in PDAC progression. Acinar gland cells **(right)** of the pancreas produce trypsin, which influences the TGFβ signaling pathway in pancreatic duct cells (left). Increased expression of cathepsin B (CTSB) in acinar gland cells will cause premature activation of trypsinogen into trypsin 1/2 serine protease 1/2 (PRSS1/2), which in turn can activate elastase 1), chymotrypsin 2), kallikrein 3), carboxypeptidase 4), phospholipase 5) and colipase (CLPS, 6). Premature activation of these serine proteases will lead to injury of pancreatic cells, pancreatitis and ultimately, pancreatic cancer. Acinar gland cells also synthesize and export SPINK1, which inhibits trypsin. Thus, in acinar gland cells, SPINK1 plays a protective role by inhibiting prematurely activated trypsin thus preventing trypsin from causing cellular damage. Premature activation of trypsinogen into trypsin triggers a signaling cascade that causes injury to pancreatic cells. In pancreatic duct cells **(left)**, trypsin is involved in the activation of coagulation factor II thrombin receptor (F2R) like trypsin receptor 1 (F2RL1). This G-protein coupled trypsin receptor influences the signaling of TGFβ receptor 2 (TGFBR2) or of Smad2/3, which is an intracellular signal transducer and transcriptional modulator activated by TGF-beta. Increased expression of TGFβ or its receptors triggers a signaling cascade via Smad2/3 processes such as epithelial mesenchymal transition (EMT), angiogenesis, and metastasis, which are associated with cancer progression.

As we supposed a pathophysiological relevance of LSBP genes that are associated with three functional annotations (Figure 5E and Supplementary Table S2) we searched by IPA software for pathways with bearing for pancreatic ductal adenocarcinoma, which contain respective LSBPs with significantly altered gene expression. Here, we identified the serine protease inhibitor Kazal-type 1 (SPINK1) pathway as most interesting and relevant with regard to pancreatic cancer development. In this pathway, cathepsin B was increased by 5 and 1.6 fold in patient and rat data, respectively, which is a key modulator triggering a cascade that causes pancreatic cancer. In addition, the activated trypsin from this pathway influences signaling of the transforming growth beta (TGFB) pathway, which in turn leads to pancreatic cancer development (Figure 7).

### Suitability of Riproximin for Treating Pancreatic Ductal Adenocarcinoma

**FIGURE 8:**
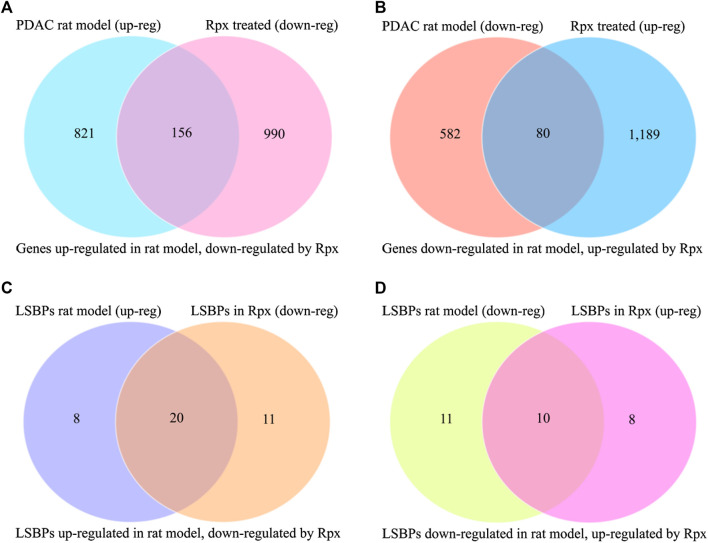
Analysis of Rpx influence on Suit2-007 genes potentially driving cancer progression. The Venn diagrams show genes, which were up-regulated in a PDAC rat progression model but down-regulated by Rpx in vitro **(A)** or vice versa **(B)**. When identifying common genes between the LSBPs derived from Supplementary Figures S1A,B, which are analyzed in Table 1 (389 genes in total), 49 LSBP genes emerged. **(C,D)** Show the respective modulation of these LSBP genes, which were up-regulated in the PDAC rat progression model but down-regulated by Rpx in vitro **(C)** or vice versa **(D)**

In order to assess the potential suitability of Rpx for treating Suit2-007 PDAC cells growing in vivo, the modulation of cancer progression genes was related to the respective effect by Rpx. From 977 genes, which showed significant up-regulation when comparing their growth in pancreatic and liver sites, 156 genes (16.0%) were significantly down-regulated by exposure to Rpx (Figure 8A). Conversely, from 662 genes, which showed significant down-regulation in the progression model, 80 genes (12.1%) were significantly up-regulated by exposure to Rpx (Figure 8B). Thus, from all 1,639 genes modulated in association with cancer progression in the rat model, 236 genes (14.4%) were modulated by Rpx in a significantly opposite way. When limiting these relations to LSBPs only, the respective gene numbers were 71% down-regulated and 48% up-regulated by Rpx (Figures 8C,D).

## Discussion

PDAC is one of the most lethal malignancies with limited options for therapy. With the current projections painting a grim picture of high mortality by 2030 (Buscail, 2017), the search for novel therapies continues. In this respect, the plant lectin riproximin is a potential candidate, as it was found effective against human and rat PDAC liver metastases in animal models (Adwan et al., 2014b; Murtaja et al., 2018). In our previous study, we described a subgroup of proteins isolated from PDAC cells, i.e., LSBPs, some of which showed significant changes in expression in both, primary and metastatic organs of an established rat model (Sagini et al., 2020). These findings gave an indication that LSBPs may have a role in metastatic progression. In this follow-up study, we investigated commonalities between Rpx and LSBPs and assessed the effect of Rpx on tumor derived LSBPs with regard to metastasis. The rationale of the study based on the same method, which had been used for isolating these proteins (Bayer et al., 2012b; Sagini et al., 2020).

Voss et al. showed that Rpx binds to galactose, a feature that led to its purification by affinity chromatography (Voss et al., 2006b). Using the same approach, we evaluated a panel of 13 monosaccharides for their affinity to the two binding sites of an Rpx homology model. In extending the findings of Voss et al., we show that Rpx does not bind to galactose only, but to all monosaccharides investigated, although with varying affinities. Of the 13 monosaccharides, open-chain rhamnose, β-d-galactose and α-l-galactopyranose bound strongest, whereas all others showed comparable affinities. Currently, the reason is unknown, why the strongest binders showed higher affinity for site 1 than for site 2 of Rpx’s B-chain. The presence of aromatic amino acids, which have an affinity for monosaccharides (Sujatha et al., 2004), cannot explain this difference, as this group was represented only in site 2, but not in site 1. Nevertheless, the binding of Rpx to these monosaccharides underscores the selectivity of this RIP with respect to cell entry. For Rpx to enter a cell, its B-chain has to interact with a myriad of glycans decorating the cell surface. This is supported by the findings of Bayer et al., which demonstrated that the two sub-domains of the Rpx B chain interact with Galβ and GalNAc moieties present on Tn antigens (Bayer et al., 2012a).

To confirm the results of the docking experiments, we evaluated the binding of Rpx to selected monosaccharides by NMR spectroscopy. For these experiments, line broadening and waterLOGSY studies confirmed the binding of d-galactose, l-fucose and l-rhamose to Rpx. The affinity of the aforementioned monosaccharides for Rpx exhibited striking similarity with that of LSBPs. In both cases, the order of binding showed preference for galactose, followed by fucose and rhamnose (Sagini et al., 2020).

By the cell proliferation assay, we also compared the activities of Rpx with LSBPs. For the first time, we show that cell lysates isolated from tumor cells inhibit cell proliferation when exposed to their cells of origin. Following successful uptake, both treatments reduced cell proliferation. The respective IC_50_s of these proteins differed by a factor of 10^4^, which is reminiscent of the differences between a purified protein and the crude extract, it was isolated from (Voss et al., 2006a). Interestingly, co-exposure of these proteins with galactose before cells’ treatment partially inhibited their activities, thus reinforcing the previous findings that these proteins are galactose binders.

To assess the effect of Rpx on LSBP genes, a chip array experiment was performed on Suit2-007 cells treated with Rpx. With IPA analysis, Rpx-modulated genes were evaluated for their respective changes in expression, in relation to other data sets from a PDAC rat model and PDAC patients. Our findings show that Rpx has the potential to decrease the expression of genes, which showed significant up-regulation in a PDAC progression rat model. When considering all genes, Rpx normalized 14% of genes modulated in the rat model of progression. The effect was more pronounced in the subgroup of LSBP genes, in which Rpx normalized 61% of those that were modulated in the rat model. This is particularly intriguing given that metastatic progression is dependent on the interaction between ligands and their respective receptors within the tumor microenvironment. As the LSBPs have been shown to bind monosaccharides, these proteins have the potential to interact with cellular glycans bearing these sugars (Nishida-Aoki and Gujral, 2019).

With the IPA program, we identified a subgroup of LSBP genes, which are associated with the terms “cell movement”, “cell signaling” and “cell death and survival”, which may contribute to metastasis. Remarkably, these genes were significantly up regulated in both, the rat progression model and PDAC patient samples. In PDAC cells treated with Rpx, some of these genes showed decreased expression compared to control samples, suggesting that they could be potential targets of this lectin. This is corroborated by our finding that the majority of these genes play a role in the ASML rat cell line, too. Furthermore, the effect of Rpx on these two cell lines was shown before (Adwan et al., 2014b; Murtaja et al., 2018). Implantation of Suit2-007 and ASML cells into the liver of rats was followed by unrestrained growth in controls, but Rpx caused regression in either model.

Among the genes, which were down regulated by Rpx, is the cathepsin family of genes (CTSB, CTSC and CTSD). These genes were significantly upregulated in the tumor progression model and in PDAC patient samples (see Figure 6). Cathepsins are ubiquitous proteases involved in regulating protein turnover, bone remodeling and keratinocyte differentiation (Singh and Saraya, 2016). In line with our study, other authors have shown that cathepsins (B and D) are deregulated in pancreatic cancer (Shen et al., 2004; Chen et al., 2007). For instance, the suppression of cathepsin B slowed down tumor progression by decreasing initiation, proliferation, angiogenesis, as well as invasion of pancreatic cancer (Gocheva et al., 2006). Cathepsin B is also a key player in the SPINK1 (serine protease inhibitor Kazal-type 1) pathway leading to pancreatic cancer (Mehner and Radisky, 2019). In the acinar gland cells of the pancreas, SPINK1 plays a protective role by inhibiting prematurely activated trypsin from causing cellular damage. In this pathway, increased expression of cathepsin B triggers a signaling cascade that damages pancreatic cells because of the premature activation of trypsinogen into trypsin. This may result in acute or chronic pancreatitis and subsequent development of pancreatic cancer. Premature activation of trypsin can also activate Smad2/3 proteins in the TGF beta signaling pathway through its elements such as coagulation factor II (thrombin) receptor like 1 (F2RL1) or transforming growth factor-β receptors 1/2 (TGFβRs 1/2). These activities may influence the development of pancreatic cancer through epithelial mesenchymal transition and angiogenesis. Remarkably, Rpx may influence the activity of these pathways by down-regulating the cathepsin family of proteins, including cathepsin B. The decreased expression of cathepsins by Rpx in Suit2-007 cells indicates that these genes are potential targets of Rpx.

Unlike cathepsins, mucins are transmembrane glycoproteins, which are also deregulated in pancreatic ductal adenocarcinoma (Besmer et al., 2011). In PDAC, mucins are involved in metabolic reprogramming of pancreatic cancer cells (Gunda et al., 2017). In the present study, we show that the MUC1 gene coding for mucin1, which was significantly up regulated in the rat progression model and PDAC patient samples, is another potential target of Rpx. Of note, mucins bears *O*- glycosylation sites, which preferentially interact with Rpx, thus enabling its entry into tumor cells (Hanisch, 2001; Bayer et al., 2012a; Madsen et al., 2012).

In conclusion, the type II ribosome inhibiting protein riproximin is a plant lectin that has been isolated by affinity chromatography from the subtropical plant *X. americana*. The same technique in combination with mass spectrometry was used for isolating and identifying cellular proteins from Suit2-007 human and ASML rat pancreatic cancer cells. These proteins were termed lactosyl-sepharose binding proteins (LSBPs) and they supposedly have significance in cancer progression in both cell lines. Both, Rpx and LSBPs exerted anti-proliferative effects in Suit2-007 cells, which were partially antagonized by co-exposure to galactose. At IC50, Rpx modulated the expression of a considerable number of all cellular genes, a property, which in all probability is part of its mechanism of action. Most remarkably, Rpx normalized the expression of a high number of LSBP genes, which may be linked to cancer progression (metastasis) by their modulated expression. This hypothesis will need, of course, further confirmation. The targeting of LSBPs by Rpx presumably relates to properties, which are based on their common affinity for sugar structures.

## Data Availability Statement

The data discussed in this publication have been deposited in NCBI's Gene Expression Omnibus (Edgar et al., 2002) and are accessible through GEO Series accession numbers GSE152304 (https://www.ncbi.nlm.nih.gov/geo/query/acc.cgi?acc= GSE152304) and GSE152305 (https://www.ncbi.nlm.nih.gov/geo/query/acc.cgi?acc= GSE152305).

## Author Contributions

MZ performed the experiments, read and approved the manuscript. MS, KK, and AO performed the experiments. MS and MB wrote the manuscript and edited it. All authors read and approved the article.

## Funding

This work was funded by the Ministry of Higher Education, Science and Technology Kenya/NACOSTI)/German Academic Exchange Service scholarship award (MSN).

## Conflict of Interest

Author AO was employed by the company MolSoft LLC.

The remaining authors declare that the research was conducted in the absence of any commercial or financial relationships that could be construed as a potential conflict of interest.
