# Supplementary material for: Riproximin Exhibits Diversity in Sugar Binding, and Modulates some Metastasis-Related Proteins with Lectin like Properties in Pancreatic Ductal Adenocarcinoma
Source: Front Pharmacol. 2020 Nov 30;11:549804. doi: 10.3389/fphar.2020.549804 (PMC7734336; doi:10.3389/fphar.2020.549804)
Supplement: Supplementary file 1 [file datasheet1.pdf]

## 1.1 Supplementary Figures

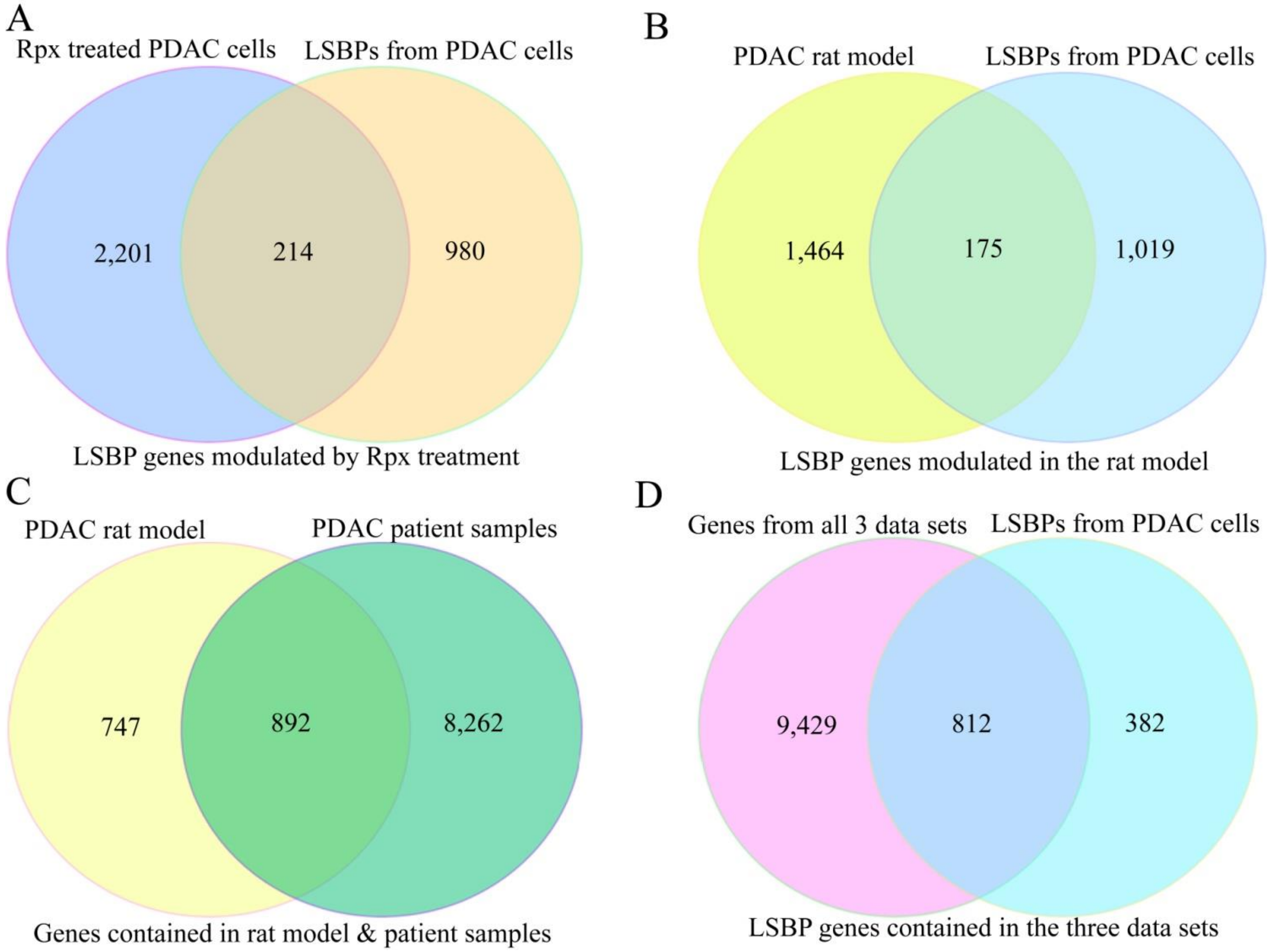


**Supplementary Figure 1:** Analysis of genes modulated in various data sets.
Figure 1A: Overlap of genes modulated in response to riproximin with those coding for lactosyl-sepharose binding proteins.
Figure 1B: Overlap of genes associated with cancer progression (rat model) with those coding for lactosyl-sepharose binding proteins.
Figure 1C: Overlap of genes associated with cancer progression (rat model) with those from patient samples.
Figure 1D: Overlap of genes combined from all three data sets with those coding for lactosyl-sepharose binding proteins.

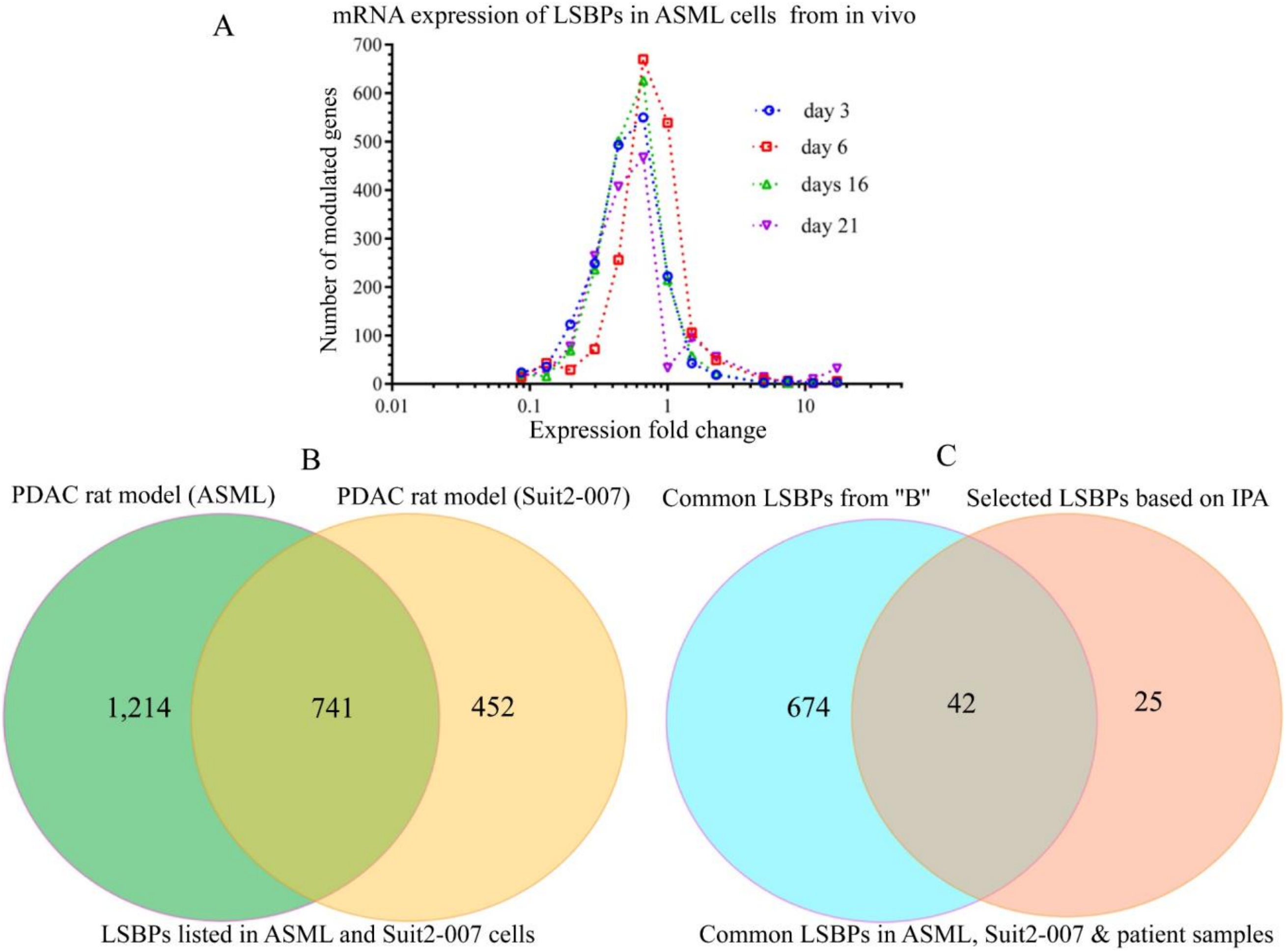


**Supplementary Figure 2:** Expression of LSBPs in rat ASML pancreatic cancer cells.

Figure 2A shows the foldchange of the mRNA of LSBP genes in ASML cells, which had been re-isolated from rat liver after 3 (blue), 6 (red), 16 (green), and 21 (purple) days. Figure 2B shows the overlap (n=741) of LSBPs found in Suit2-007 cells (yellow circle) and ASML cells (green circle). Figure 2C shows a Venn diagram of LSBP genes, which were present both, in Suit2-007 and ASML cells, and genes selected for their functional annotation from Suit2-007 cells. The overlap of 42 LSBPs corresponds to those proteins which are related to the functional annotations ‘cell movement’, ‘cell signaling’ and ‘cell death and survival’, in both cell lines.

**Supplementary Table 1 A: Docking values for binding site 1**

| Molecule | Score | N-atom | N-flex | H-bond | H-phob | V-wInt | E-intl | D-solv | Sol-El | Mf-Score | dTSsc |
|---|---|---|---|---|---|---|---|---|---|---|---|
| α-D-galactose | -8.57 | 24 | 6 | -5.3 | -1.7 | -10.33 | 0.00 | 11.25 | 6.47 | -35.68 | 0.71 |
| β-D-galactose | -14.96 | 24 | 6 | -8.98 | -1.2 | -12.64 | 4.36 | 17.39 | 6.9 | 2.78 | 0.62 |
| α -L-rhamnose | -6.8 | 23 | 4 | -4.01 | -1.82 | -10.26 | 0.79 | 12.8 | 4.27 | 7.76 | 0.78 |
| β -L-rhamnose | -9.5 | 23 | 4 | -4.99 | -1.79 | -10.94 | 1.64 | 13.52 | 4.11 | -3.18 | 0.58 |
| α -D-fucose | -9.6 | 23 | 4 | -5.38 | -1.35 | -11.15 | 0.82 | 13.93 | 4.86 | 4.00 | 0.88 |
| β -D-fucose | -12.47 | 23 | 4 | -7.19 | -1.39 | -11.91 | 2.37 | 15.25 | 7.15 | -0.37 | 0.61 |
| α -L-galactopyranose | -13.72 | 24 | 6 | -7.47 | -1.64 | -9.82 | 0.00 | 12.64 | 4.56 | 9.78 | 0.62 |
| β -L-galactopyranose | -8.93 | 24 | 6 | -6.62 | -1.32 | -10.33 | 2.15 | 13.95 | 7.51 | 11.64 | 0.65 |
| D-mannomethylose | -8.73 | 23 | 4 | -4.47 | -1.57 | -12.44 | 0.2 | 13.95 | 4.85 | 3.83 | 0.83 |
| α -L-fucose | -9.64 | 23 | 4 | -4.69 | -1.93 | -11.07 | 1.08 | 11.33 | 5.6 | 3.24 | 0.59 |
| β –L-fucose | -8.3 | 23 | 4 | -6 | -1.33 | -8.63 | 2.04 | 15.2 | 3.97 | 3.07 | 0.51 |
| galactose open | -5.77 | 24 | 10 | -6.11 | -1.27 | -9.74 | 6.24 | 19.12 | -0.1 | 3.1 | 0.74 |
| rhamnose open | -17.13 | 23 | 8 | -7.28 | -1.43 | -14.39 | 3.1 | 12.78 | 3.4 | -3.15 | 0.78 |

**Supplementary Table 1 B Docking values for binding site 2**

| Molecule | Score | N-atom | N-flex | H-bond | H-phob | V-wInt | E-intl | D-solv | Sol-El | Mf-Score | dTSsc |
|---|---|---|---|---|---|---|---|---|---|---|---|
| α-D-galactose | -7.62 | 24 | 6 | -3.52 | -1.76 | -9.34 | 0 | 8.91 | 2.32 | -46.68 | 0.47 |
| β-D-galactose | -11.72 | 24 | 6 | -4.27 | -1.65 | -11.82 | 0.17 | 8.68 | 2.86 | -33.52 | 0.59 |
| α -L-rhamnose | -11.28 | 23 | 4 | -3.37 | -2.04 | -10.26 | 0.9 | 6.66 | 2.23 | -38.53 | 0.59 |
| β -L-rhamnose | -10.9 | 23 | 4 | -3.32 | -1.97 | -10.61 | 0.43 | 8.84 | 0.58 | -38.31 | 0.65 |
| α -D-fucose | -10.5 | 23 | 4 | -2.83 | -1.67 | -8.71 | 0 | 5.47 | -0.04 | -35.04 | 0.44 |
| β -D-fucose | -13.54 | 23 | 4 | -3.49 | -2 | -11.4 | 0.06 | 5.61 | 2.17 | -35.6 | 0.57 |
| α -L-galacto-pyranose | -11.21 | 24 | 6 | -3.99 | -1.78 | -9.53 | 0 | 6.35 | 1.89 | -39.7 | 0.67 |
| β -L-galacto-pyranose | -10.32 | 24 | 6 | -3.83 | -1.55 | -9.46 | 0.18 | 8.32 | 0.05 | -34.17 | 0.71 |
| D-manno-methylose | -8.92 | 23 | 4 | -3.87 | -1.8 | -9.61 | 2.03 | 8.92 | 3.92 | -38.12 | 0.54 |
| α -L-fucose | -11 | 23 | 4 | -3.02 | -2.23 | -9.74 | 0.15 | 5.69 | 1.82 | -37.07 | 0.62 |
| β -Lfucose | -7.3 | 23 | 4 | -2.91 | -1.82 | -9.26 | 1.54 | 8.95 | 2.15 | -32.9 | 0.6 |
| galactose-open | -7.03 | 24 | 10 | -3.57 | -1.57 | -9.46 | 0 | 10.12 | -1.65 | -34.47 | 0.74 |
| rhamnose open | -11.63 | 23 | 8 | -4.39 | -1.75 | -10.47 | 5.01 | 9.67 | -1.41 | -35.44 | 0.56 |

**Score:** is the overall docking score (see ICM User's Guide). A negative score is a good indication that a ligand might bind.

**Hbond**: is Hydrogen Bond energy, **Hphob**: is the hydrophobic energy in exposing a surface to water

**VwInt**: is the van der Waals interaction energy (sum of gc and gh van der Waals). Current version of the score uses explicit van der Waals interaction energy calculation (no grids)

**Eintl:** is internal conformation energy of the ligand, **Dsolv**: is the desolvation of exposed h-bond donors and acceptors

**SolEl:** is the solvation electrostatics energy change upon binding, **Mfscore:** is the mean force score

**Nflex:** is the number of rotatable torsions, **dTSc:** Loss of entropy by the rotatable protein side-chains.

**Supplementary Table 2: Venn diagram analysis for lactosylsepharose binding proteins involved in selected functional annotations in patients, rat model and riproximin treated samples**

| Gene symbol | Gene description for respective lactosylsepharose binding proteins | Patient samples (FC) | PDAC rat model Liver / pancreas (FC) | Rpx treated PDAC cells (FC) |
|---|---|---|---|---|
| **a) One functional annotation - Cell movement** | | | | |
| ACTN1* | Actinin alpha 1 | 3.10 | 0.66 | 1.03 |
| CTSC* | Cathepsin C | 4.94 | 1.61 | 0.73 |
| DPYSL2* | Dihydropyrimidinase like 2 | 3.59 | 1.53 | 0.44 |
| EPCAM | Epithelial cell adhesion molecule | 1.29 | 1.75 | 0.62 |
| HOMER3* | Homer scaffold protein 3 | 1.87 | 0.62 | 1.51 |
| **b) One functional annotation- Cell signaling** | | | | |
| PSMB9* | Proteasome subunit beta 9 | 4.50 | 1.66 | 0.67 |
| FGG | Fibrinogen gamma chain | 0.66 | 106.47 | 1.04 |
| PPP1R18 | Protein phosphatase 1 regulatory subunit 18 | 2.96 | 0.67 | 2.19 |
| **c) One functional annotation - Cell death and survival** | | | | |
| RBBP4* | RB binding protein 4, chromatin remodeling factor | 1.29 | 1.55 | 0.47 |
| PRDX2* | Peroxiredoxin 2 | 0.62 | 1.49 | 0.63 |
| TMED10 | Transmembrane p24 trafficking protein 10 | 0.66 | 1.61 | 0.65 |
| FHL1* | Four and a half LIM domains 1 | 1.96 | 0.59 | 0.66 |
| PABPC1* | Poly(A) binding protein cytoplasmic 1 | 2.42 | 1.46 | 0.68 |
| FAIM | Fas apoptotic inhibitory molecule | 0.72 | 1.63 | 0.67 |
| LAMP2 | Lysosomal associated membrane protein 2 | 2.13 | 1.78 | 0.69 |
| HNRNPA1 | Heterogeneous nuclear ribonucleoprotein A1 | 2.58 | 1.74 | 0.89 |
| FTH1* | Ferritin heavy chain 1 | 1.68 | 1.82 | 0.89 |
| XRCC6* | X-ray repair cross complementing 6 | 1.51 | 0.66 | 1.13 |
| TXNRD1* | Thioredoxin reductase 1 | 2.72 | 0.64 | 2.10 |
| LMNA* | Lamin A/C | 3.41 | 0.46 | 0.97 |
| EFEMP1 | EGF containing fibulin ECM protein 1 | 5.14 | 1.83 | 1.09 |
| SLK* | STE20 like kinase | 2.76 | 0.59 | 1.14 |
| EIF4G2* | Eukaryotic translation initiation factor 4 γ2 | 0.49 | 1.67 | 1.28 |
| ALDOA* | Aldolase, fructose-bisphosphate A | 2.28 | 1.81 | 0.85 |

| | | | | |
|---|---|---|---|---|
| COMT* | Catechol-O-methyltransferase | 1.82 | 0.54 | 0.95 |
| DYNC1H1 | Dynein cytoplasmic 1 heavy chain 1 | 2.19 | 0.58 | 1.23 |
| MCM2* | Minichromosome maintenance complex component 2 | 2.19 | 0.68 | 0.89 |
| CHMP5* | Charged multivesicular body protein 5 | 2.57 | 2.26 | 1.44 |
| RBM3* | RNA binding motif protein 3 | 1.11 | 0.64 | 1.55 |
| SPTBN1* | Spectrin beta, non-erythrocytic 1 | 1.74 | 1.80 | 1.60 |
| HMGA1 | High mobility group AT-hook 1 | 2.19 | 0.53 | 1.64 |
| EIF6* | Eukaryotic translation initiation factor 6 | 1.62 | 0.65 | 1.78 |
| PSMD2* | Proteasome 26S subunit, non-ATPase 2 | 2.05 | 0.68 | 2.17 |
| MAP1LC3B* | Microtubule associated protein 1 light chain 3 beta | 1.27 | 1.71 | 2.39 |
| **d) Two functional annotations -Cell movement &cell signaling** | | | | |
| CD276 | CD276 molecule | 1.63 | 0.59 | 0.61 |
| VASP* | Vasodilator stimulated phosphoprotein | 2.19 | 0.51 | 1.21 |
| **e) Cell signaling and cell death and survival** | | | | |
| IFI16 | Interferon gamma inducible protein 16 | 6.93 | 1.63 | 1.08 |
| FSTL1 | Follistatin like 1 | 5.09 | 1.69 | 0.77 |
| PKM* | Pyruvate kinase M1/2 | 3.76 | 0.51 | 0.93 |
| KRT18 | Keratin 18 | 1.00 | 1.90 | 1.15 |
| FNTA* | Farnesyltransferase, CAAX box, alpha | 0.82 | 1.55 | 2.04 |
| TFAM | Transcription factor A, mitochondrial | 0.73 | 0.68 | 1.29 |
| VIM* | Vimentin | 7.25 | 0.68 | 1.05 |
| **f) Cell movement and cell death and survival** | | | | |
| CRK* | CRK proto-oncogene, adaptor protein | 0.80 | 0.67 | 1.75 |
| CTSB* | Cathepsin B | 5.19 | 1.62 | 0.44 |
| MSN* | Moesin | 3.89 | 0.56 | 1.02 |
| TGFBI | Transforming growth factor beta induced | 5.23 | 1.58 | 0.76 |
| **g) Three functional annotations-cell movement, signalling, and death & survival** | | | | |
| ANXA1* | Annexin A1 | 14.04 | 1.78 | 1.16 |
| PLAU | Plasminogen activator, urokinase | 9.81 | 0.53 | 1.26 |
| SERPINE2 | Serpin family E member 2 | 4.82 | 2.324 | 0.71 |
| ICAM1 | Intercellular adhesion molecule 1 | 4.67 | 0.55 | 0.88 |
| TGM2* | Transglutaminase 2 | 4.42 | 0.63 | 0.72 |
| LGALS3 | Galectin 3 | 3.61 | 1.59 | 0.87 |

| | | | | |
|---|---|---|---|---|
| GRN | Granulin precursor | 3.40 | 1.57 | 0.61 |
| HLA-A | Major histocompatibility complex, class I, A | 3.38 | 1.75 | 1.31 |
| ANXA2* | Annexin A2 | 3.13 | 2.08 | 3.75 |
| C3* | Complement C3 | 2.96 | 1.54 | 1.10 |
| IL6ST | Interleukin 6 signal transducer | 1.94 | 1.46 | 2.04 |
| EGFR* | Epidermal growth factor receptor | 0.52 | 0.68 | 2.08 |
| **h) LSBP genes that were not associated with functional annotations** | | | | |
| CTSD* | Cathepsin D | 2.90 | 1.69 | 0.66 |
| PGK1* | Phosphoglycerate kinase 1 | 2.81 | 1.42 | 0.68 |
| GNS* | Glucosamine (N-acetyl)-6-sulfatase | 2.77 | 1.64 | 0.70 |
| HNRNPA2B1* | Heterogeneous nuclear ribonucleoprotein A2/B1 | 4.40 | 1.77 | 0.75 |
| ADD3* | Adducin 3 | 2.33 | 1.79 | 0.45 |
| RCN2* | Reticulocalbin 2 | 2.51 | 1.37 | 0.58 |
| MUC1 | Mucin 1, cell surface associated | 3.31 | 1.20 | 0.59 |
| TIMP2 | TIMP metallopeptidase inhibitor 2 | 4.55 | 1.33 | 0.63 |

* The overlapping genes between Suit2-007 and ASML cells are marked with an asterisk.
